# Integrating renewable energy devices with streetscape elements to electrify the Egyptian roads

**DOI:** 10.1038/s41598-023-32773-4

**Published:** 2023-04-25

**Authors:** Rania Rushdy Moussa, Marianne Nabil Gurguis

**Affiliations:** Architectural Engineering Department, The British University in Egypt, El-Sherouk City, Egypt

**Keywords:** Sustainability, Energy science and technology, Renewable energy

## Abstract

The high percentage of carbon emissions, which leads to various environmental problems such as air pollution and global warming, is one of the critical issues resulting from the growth of cities. International agreements are being established to prevent these negative effects. Non-renewable resources are also being depleted and may become extinct in future generations. Due to the extensive use of fossil fuels by automobiles, data show that the transportation sector is responsible for roughly a quarter of worldwide carbon emissions. On the other hand, in developing nations, energy is scarce in many neighborhoods and districts because the governments are unable to meet the community's need for power supply. This research aims to work on techniques that will reduce the carbon emissions produced by roadways while also building environmentally friendly neighborhoods by electrifying the roads using (RE). A novel component called "Energy-Road Scape" (ERS) elements will be used to demonstrate how to generate (RE) and, hence, reduce carbon emissions. This element is the result of integrating streetscape elements with (RE). This research presents a database for ERS elements and properties as a tool for architects and urban designers to design ERS elements instead of utilizing regular streetscape elements.

## Introduction

Carbon dioxide emissions have been increasing excessively since the industrial revolution^1^.

The industrial revolution changed our planet, it speeded up life as a positive outcome, but on the other hand, it started to create a load of carbon dioxide emissions that became a problem as a negative outcome. Nowadays, it is not only a problem, but a threat to life on planet earth with its natural balance of the ecosystem^2^, because it is one of the major constituents of the greenhouse gas emissions causing climatic change^19,20^.

Thus, the research community has a viable task of performing both corrective and preventive measures. The corrective actions start by improving and developing operations of filtration and purifying of the air and water of our planet. However, only preventive measures are to crown any corrective measures with success^3^. The reason for that is if the threatening reasons persist, then, corrections will be definitely ineffective. Therefore, the most important task is to eliminate the reasons for this blow of carbon emissions on earth. According to the EIA, the first reason for Carbon emissions resulting from fuel combustion is electricity and heat, while the second one is the resultant exhaust from transportation. These two reasons add up collectively to about 65%^4^.

This research addresses both aspects in an attempt to prevent and eliminate causes that lead to increase in carbon emissions. The novelty of this study is presenting new elements called “Energy-RoadScape” (ERS). These elements integrate RE devices with streetscape elements to generate clean energy and create sustainable neighborhoods. This research intends to reduce the amount of energy taken from the governments of developing countries, which are already facing shortage in their base needs for energy supply. In addition, using ERS elements will impact the neighborhoods’ economic, social, public health, and environmental stated, as well as it will slow the spread of global issues like carbon emissions, ozone depletion, and climate change which is produced from generating energy using fossil fuel.

## Hypothesis

The hypothesis of this research is that the high level of carbon emissions produced from roads is from electricity production and the heavy use of vehicles and tucks for fossil fuels. Integrating Streetscape elements with RE sources will create Enery-RoadScape elements, in which it will produce clean and efficient energy that can be used for lighting the neighbourhood’s streets and could be used to generate electricity for electric spencer that charge the electric vehicles.

## Literature review

### Streetscape elements

**Figure 1:**
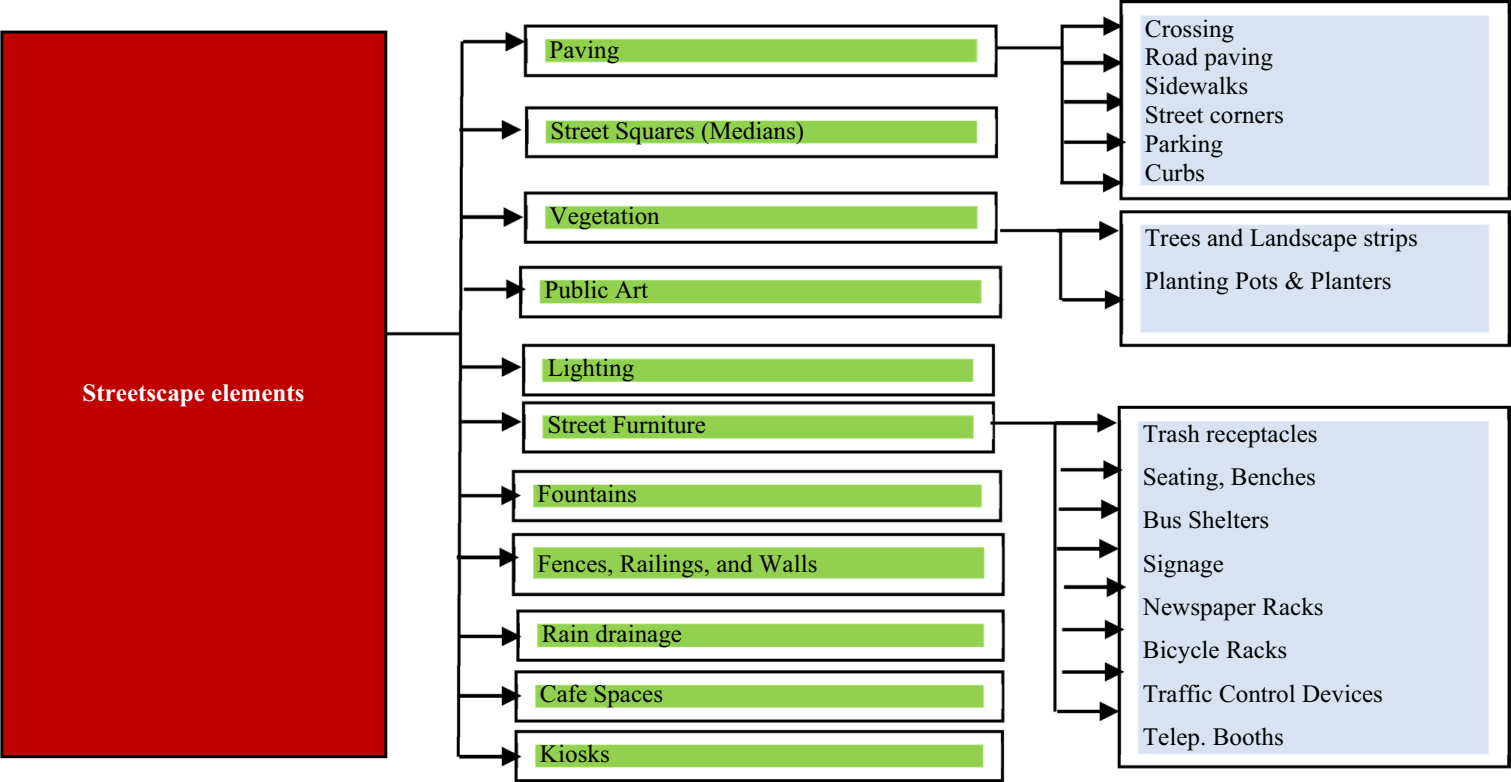
Streetscape elements^5^.

To retain the aesthetic and functional qualities of the streetscape, different streetscape fittings are positioned in public or communal areas^5^. The impact of these elements on human health, social life, economy, environment, and psychology of human actions was discovered by researchers through their research path^6^, which is why many streetscape designers, architects, and urban designers regarded them as aesthetic, healthy, and special features in their designs^7^. This encouraged individuals to consider the significance of streetscape components deeply^8^. Various academics have categorized the streetscape components in various ways over time, the components shown in Fig. 1 made up the framework of streetscape elements employed in this study. This framework lists all the components that have previously been discussed by various researchers.

### Renewable energy (RE) sources

**Figure 2:**
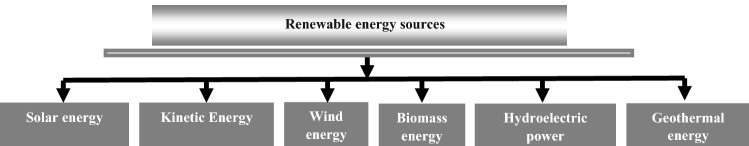
Renewable energy sources.

Human efforts to use (RE) sources have altered because of the current energy crisis^9^. The industry for (RE) has shown tremendous technological advancement during the past fifteen years^10^. The rising level of competition with conventional power plants provided momentum to the global market for (RE) technology^11^. According to previous research statistics within the field of (RE), the use of sun, wind, hydropower, geothermal, and biomass can generate enough energy to serve the world's population more than a few times over^12^. However, this is considered anticipatory if compared to the actually practical usage. In fact, few technologies for harnessing (RE) have proven to be effective, while the economic viability of this technology is restricted to a small number of countries worldwide^13^. Markets are frequently drastically changed by grants in favour of fossil fuels, which push (RE) to the back burner^14^. Early "entrance into the (RE) technologies market, aimed to start a transformation to an energy system based on (RE) technologies and building of production capacities and installations of (RE) technologies, and benefit from their macroeconomic development^15^. Additionally, as more technological advancements are made, (RE) technologies are becoming competitive without investment support^16^. As shown in Fig. 2, this study will describe the characteristics, installation methods, and materials used for (RE) sources such solar energy, kinetic energy, wind energy, biomass energy, hydroelectric power, and geothermal energy.

## Methodology

This research consists of two parts: theoretical and empirical. The theoretical part presents streetscape elements and introduces (RE) sources. The empirical part presents the properties of new sustainable elements called Energy-RoadScape elements, which integrate (RE) devices with streetscape elements as a tool for reducing CO2 emissions from roads. In which it will solve the infrastructure problem facing the spreading of the electric vehicle industry in developing nations. The research will present a database to help the designers calculate the amount of energy produced from using ERS as well as the properties of each element. The properties of the ERS elements are divided into the initial cost of installing this element, the area needed to operate efficiently, and the life-time and annual maintenance cost of each element.

## Empirical work

**Figure 3:**
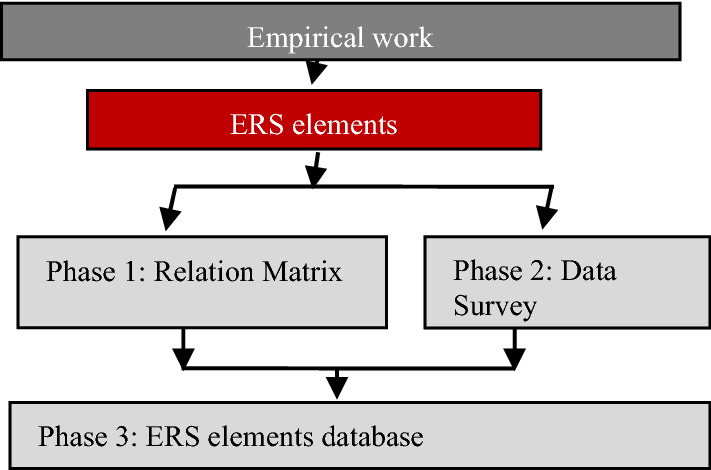
Empirical framework.

The goal of the research is to identify the Energy-RoadScape ERS) Elements and properties, which result from integrating streetscape elements with (RE) devices. The characteristics of the ERS elements are identified, analyzed, and presented in three phases as shown in Fig. 3.

### Phase one: relation matrix

The relation matrix identifies the streetscape elements such as sidewalks, trees and landscape strips, etc. that can be integrated with (RE) devices, such as solar, wind, biomass, hydropower, kinetic energy, and geothermal energy generators. The results of this phase is a new elements called ERS. Depending on previous studies and real-life applications that integrated Streetscape elements with (RE) devices, this research created a relation matrix as illustrated in Table 1.

**Table 1:**
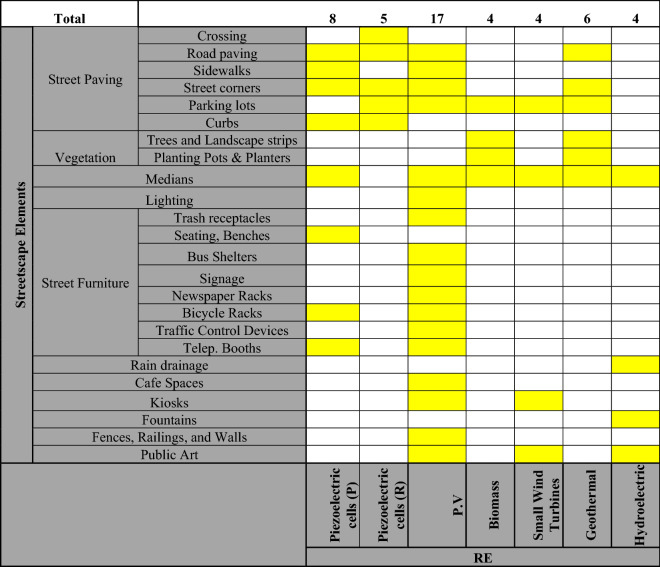
Relation matrix between streetscape elements and RE.

This research was able to identify the categories of streetscape features that can be integrated with RE to create new elements called (ERS). This study focused on the small types of renewables that can be integrated with streetscape elements such biomass, kinetic energy (piezoelectric cells), PV, tiny hydropower plants, geothermal, and small wind turbines that can be installed in small spaces. Massive wind turbines and enormous hydropower plants were excluded from this study due to their excessively large footprints. Table 1, shows that we can create forty-eight ERS elements, which is resulted from integrating 11 main streetscape elements and 13 sub-elements (for a total of 24 streetscape elements) with 7 RE sources. This is the number of possibilities resultant from the integration of streetscape elements with RE devices in the matrix. This will give streetscape designers, architects, landscapers, urban designers, and transportation engineers a variety of options during the designing process of their projects using ERS elements. The table also showed that P.V. cells can be integrated with 17streetscape elements, which is considered the most significant energy sources that can be integrated with streetscape elements. On the other hand, Geothermal and Piezoelectric (Pavement) cells create 6 and 8 ERS elements, respectively.

### Phase two: cross-sectional survey

**Figure 4:**
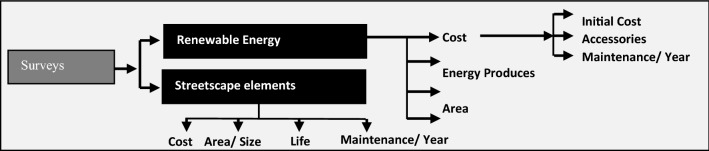
The aspects of the two cross-sectional surveys’ used in this research.

The Cross-sectional surveys are used to gather data about the characteristics of RE devices and streetscape elements. In cross-sectional surveys, questionnaires are used to gather data from a population or a representative sample about a specific subject at a specific time period^17^. In this research, the data was gathered from Egyptian firms that are specialized in constructing streetscapes elements such as “Curve Streetscape Company” and RE contactors such as “I-Solar Company”, “3 M company”, Three Brothers Company, etc. Since there is still a scarcity in utilizing some types of RE devices in Egypt. For example, there is no local companies installing kinetic energy harvesting systems such as piezoelectric cells in Egypt. Hence, popular online marketing websites such as Alibaba and Amazon were employed to gather information on these materials. In this study, two cross-sectional surveys were conducted: The first cross-sectional survey focused on collected data regarding streetscape elements. While the second cross-sectional survey, was targeting RE devices as shown in Fig. 4. As indicated in Fig. 4, the first cross-sectional survey focused on the price, lifespan, energy produced, area used, and annual maintenance cost of each RE device. Since no local company operates in all RE disciplines, the data was gathered from a number of international and local companies. The second cross-sectional survey provided information on the characteristics of each streetscape element, including size, cost, lifespan, and annual maintenance costs. It was discovered that RE devices are very expensive. Using ERS elements, will increase the initial cost of installing streetscape elements almost five times more than using regular elements.

#### Cross-sectional surveys on renewable energy (RE)

A number of meetings were held with twelve directors from various Egyptian corporations to get information on the characteristics of (RE) devices between May 2022 and October 2022. A list of data summarized the characteristics of each RE device available in the companies, was provided to the research. The data provided by the managers was gathered and summarized in Table 2) to include: The initial cost of each device, the cost of accessories needed to operate that device effectively, the cost of annual maintenance, the amount of energy produced, the area needed for this device to produce the previous amount of energy, and the lifetime of this device before it needs to be replaced. In order to fill-in the data gaps for some devices, such as piezoelectric cells, which are not widely used in Egypt, a search was conducted through the online marketing sites such as Alibaba and Amazon websites. The amount of energy presented in Table 2 is correlated with the area and cost of the RE devices. Thus, if it is required to produce twice the amount of energy, the area and cost will be doubled.

**Table 2:** Data collected from cross-sectional surveys on the properties of renewable energy.

R.E source	Renewable energy (RE) properties								
	Cost				Energy produced		Area		Lifetime/year
	Initial cost	Accessories		Maintenance/year	Power	Energy/day	Size	Weight	
P.V	$850	Battery	$840	$85	300 W = 0.3 kW	0.3 × 12 h = 3.6 kWh	2 m^2^	26 kg	25
		Inverter	$27						
		Cables	$15						
Biomass	$90,409.0			$40	510 kWh		2.5 m^3^		25
Small wind turbines	$1000	Transformer	$150	$20	300 W = 0.3 kW	0.3 × 15 h = 4.5 kWh	ф1230 × 1090 mm	27 kg	20
		Capacitor	$10						
Small hydro power	$1000	Battery	$840	$50	24–800 kW		Design head = 50–5000 m	Runner diameter = 0.4–1.1 m	50
		Inverter	$27						
		Cables	$30						
Piezoelectric cells (Roads)	$6500	Battery	$840	$20	16 kWh		10 m		10–20
		Inverter	$27						
		Cables	$30						
Piezoelectric cells (Pavement)	$1500	Battery	$840	$20	2 kWh		10 m^2^		10–20
		Inverter	$27						
		Cables	$30						

In comparison to the energy produced by piezoelectric cells for roads, Table 2 demonstrates that the initial cost of piezoelectric cells is very expensive. Due to the dust and the climate in Egypt which is dominated by desert, P.V. cells require more maintenance each year than any other type of equipment. Although the small hydropower system has a similar initial cost to small wind turbines, but it generates more electricity overall.

#### Cross-sectional surveys on streetscape

In April 2022, an interview was conducted with “Curve” Landscape Company's general and financial managerS. 38 questions were posed to the head manager of the Company, 34 of which concerned the costs and characteristics of streetscape elements, that the company had previously built, and 4 of which reiterated the company history. The information in Table 3, provides an overview of the many characteristics of streetscape elements, which include product description, area, cost, lifetime, and annual maintenance. The information includes each element's local startup and ongoing repairs costs, as well as the space required to install it and its lifetime.

**Table 3:** Properties of streetscape element from data survey (source: by authors).

		International consultant				
Streetscape elements		Material name & description	Properties			
			Cost	Maintenance/year	Lifetime/year	Size
Street paving	Crossing	(OEM)cargo trail	$1000–6000	2$	25	2400 × 6000 mm
	Road paving	(OEM)cargo trail	$1000–6000	2$	15	2400 × 6000 mm
	Sidewalks	Natural granite pavement	5m^2^	2$	15	10 × 20 × 5/10 cm
	Street corners	(OEM)cargo trail	$1000–6000	2$	25	2400 × 6000 mm
	Parking lots	(OEM)cargo trail	$1000–6000	2$	50	2400 × 6000 mm
	Curbs	Natural granite pavement	5 m^2^	2.5$	25	10 × 20 × 5/10 cm
Vegetation	Trees & Streetscape strips	Natural grass for garden home lawn	$1–12/m^2^	4$	70	Customized
	Planting Pots & Planters	Natural grass for garden home lawn	$1–12/m^2^	4$	70	Customized
Medians		Natural grass for garden home lawn	$1–12/m^2^	4$	100	Customized
Lighting + solar cells		High quality new upgraded all in one energy saving high brightness solar streetlight	300	1$	25	Customized
Street furniture	Trash receptacles	Custom made stainless steel trash can/street garbage bin/outdoor waste container	$1.5–3.5	0.25$	10	Customized
	Seating, benches	Durable Steel Park bench/outdoor metal bench/Patio metal bench	$100–200	1$	10	1520 × 650 × 810mm
	Bus shelters	Modern City Bus Shelter With Solar Power System And LED Scrolling Light Box	$600–900/Unit	1.5$	25	12,070 × 3050 × 1400 mm
	Signage	Standing Advertising Aluminum Street Prisma Signage	$60–120/m^2^	0.04$	15	Customized
	Newspaper racks	Flooring acrylic outdoor newspaper racks	$50	2$	25	280 × 320 × 800 mm
	Bicycle racks	Modular bicycle parking rack	$4–6.5	2$	25	400 × 152 × 220 mm
	Traffic control devices	Traffic Control Devices	$105.0	4$	10	200 mm
	Telep. Booths	Traffic police booth kiosk booth/container house	100	3$	10	1.2 × 1.5 × 2.4 m
Rain drainage		Outdoor Rain Storm Composite Water Grates for Drainage	US $20	6$	30	400 × 600
Cafe spaces		Cafe Spaces	$1–12/m^2^	2$	40	Customized
Kiosks		Shopping mall kiosk mobile kiosk design with 3D kiosk design	$150–500/m^2^	3$	15	Customized
Fountains		Garden Water Fountain Outdoor Fountain Led Garden Fountain	2000	12$	50	Customized
Fences, railings, and walls		Q110969 artificial plant wall garden decor streetscape plants artificial living wall	$75–200 m^2^	5$	70	Customized
Public art		Public artworks	1000	8$	50	Customized

### hase three: energy-roadscape (ERS) elements database

The ERS elements database was created using the information gathered from relational matrix and the two cross-sectional surveys. The area of RE devices and the area of streetscape elements were compared in order to recalculate the properties of ERS elements. The area of streetscape elements was used as the smallest area that could be used for this integration, and the researchers recalculated the cost and the power generated for the larger area. The cost of ERS elements were calculated by sum the initial costs of the streetscape elements, the initial costs of the RE device, and the costs of the accessories required to use this device. While the cost of annual maintenance was calculated by adding the cost of the annual maintenance of RE device and streetscape elements. Regarding the lifetime, the research compared the lifetime of streetscape elements to the RE device and chose whichever is less. Table 4 lists all the characteristics of ERS elements cost, minimum area needed, daily energy production, annual maintenance cost, and lifetime. The results suggest that ERS elements will generate energy effectively for at least ten years, but it will cost more than typical streetscape elements by an average of ten times more than the regular initial cost. The annual maintenance cost of ERS elements ranges from 21 dollars at the lowest end to 93 dollars at the most. Piezoelectric cells (pavements) provide the least amount of energy per day for ERS elements; they create 2 kWh for a 10 m^2^ area, whereas geothermal energy produces 24 kWh for a 1.5 m^2^. Although geothermal energy is an uncommon source of energy and doesn't occur in most locations, it can be considered the element that produces the most energy per day.

**Table 4:** Properties of energy-RoadScape elements ERS elements.

ERS elements			ERS elements properties				
Streetscape elements		Renewable energy (RE) devices	Total cost	Area	Energy/day	Maintenance cost/year	Life time (years)
Street paving	Crossing	Piezoelectric cells (R)	$7,397	10 m	16 kWh	$22	15
	Road paving	Piezoelectric cells (P)	$2,397	10 m^2^	2 kWh	$22	15
		Piezoelectric cells (R)	$7,397	10 m	16 kWh	$22	15
		P.V	$1,871	2m^2^	3.6 kWh	$87	15
		Geothermal	$1,780,105	1.5 m^2^	24 kWh	$52	15
	Sidewalks	Piezoelectric cells (P)	$2,397	10 m^2^	2 kWh	$22	15
		P.V	$1,742	2 m^2^	3.6 kWh	$87	15
	Street corners	Piezoelectric cells (P)	$2,397	10 m^2^	2 kWh	$22	15
		Piezoelectric cells (R)	$7,397	10 m	16 kWh	$22	15
		P.V	$1,871	2 m^2^	3.6 kWh	$87	25
		Geothermal	$1,780,105	1.5 m^2^	24 kWh	$52	25
	Parking lots	Piezoelectric cells (R)	$7,397	10 m	16 kWh	$22	15
		P.V	$1,871	2 m^2^	3.6 kWh	$87	25
		Biomass	$90,583	2.5m^3^	510 kWh	$42	26
		Small Wind Turbines	$1,993	12 m^2^	4.5 kWh	$22	20
		Geothermal	$1,780,105	1.5 m^2^	24 kWh	$52	25
	Curbs	Piezoelectric cells (P)	$2,397	10 m^2^	2 kWh	$22.5	15
		Piezoelectric cells (R)	$7,397	10 m	16 kWh	$22.5	15
Vegetation	Trees and Landscape strips	Biomass	$90,424	2.5m^3^	510 kWh	$44	26
		Geothermal	$1,780,009	1.5 m^2^	24 kWh	$54	25
	Planting pots & planters	Biomass	$90,424	2.5m^3^	510 kWh	$44	26
		Geothermal	$1,780,009	1.5 m^2^	24 kWh	$54	25
Medians		Piezoelectric cells (P)	$2,397	10 m^2^	2 kWh	$24	15
		P.V	$1,744	2 m^2^	3.6 kWh	$89	25
		Biomass	$90,424	2.5m^3^	510 kWh	$44	26
		Small Wind Turbines	$1,232	ф12m^2^	4.5 kWh	$24	20
		Geothermal	$1,780,009	1.5 m^2^	24 kWh	$54	25
		Hydroelectric	$1,903	ф1.1 m	25 kWh	$54	50
Lighting		P.V	$300	2 m^2^	3.6 kWh	$86	25
Street furniture	Trash receptacles	P.V	$1,742	2 m^2^	3.6 kWh	$85.25	10
	Seating, Benches	Piezoelectric cells (P)	$2,397	10 m^2^	2 kWh	$21	10
	Bus Shelters	P.V	$1,803	2 m^2^	3.6 kWh	$86.5	25
	Signage	P.V	$1,852	2 m^2^	3.6 kWh	$85.04	15
	Newspaper Racks	P.V	$2,848	2 m^2^	3.6 kWh	$87	25
	Bicycle Racks	Piezoelectric cells (P)	$2,397	10 m^2^	2 kWh	$22	15
		P.V	$1,864	2 m^2^	3.6 kWh	$87	25
	Traffic control devices	P.V	$1,837	2 m^2^	3.6 kWh	$89	10
	Telep. booths	Piezoelectric cells (P)	$2,397	10 m^2^	2 kWh	$23	10
		P.V	$779.4	1.8m^2^	1.62kWh	$88	10
Rain drainage		Hydroelectric	$1,917	ф1.1 m	25 kWh	$56	30
Cafe spaces		P.V	$1,744	2 m^2^	3.6 kWh	$87	25
Kiosks		P.V	$2,032	2 m^2^	3.6 kWh	$88	15
		Small Wind Turbines	$1,310	ф12m^2^	4.5 kWh	$23	15
Fountains		Hydroelectric	$3,897	ф1.1 m	25 kWh	$62	50
Fences, railings & walls		Biomass	$90,484	2.5m^3^	510 kWh	$45	26
Public art		P.V	$2,732	2 m^2^	3.6 kWh	$93	25
		Small Wind Turbines	$2,160	ф12m^2^	4.5 kWh	$28	20
		Hydroelectric	$2,897	ф1.1 m	25 kWh	$58	50

## Discussion

Energy-RoadScape (ERS) is a sustainable element and it could be considered as the new generation of Streetscape elements, it is the product of integrating streetscape elements with (RE) devices. ERS share the benefit of streetscape elements and (RE) device. It affects the social state by increase the quality of life in the city offering more job opportunities, developing the community, and giving it its identity, they also help in making the neighbourhoods more liveable place; they give the families and children a place to express their feelings and an outdoor space to play and enjoy their time^18^. in addition, ERS affect the economic state by increasing the property values since that people prefer to buy a property in a sustainable city and it reduce the electricity bills in public facilities due to the usage of (RE)^7^. Moreover, ERS reduced the environmental negative impact for energy generation since fossil fuels will not burn to generate energy which will reduce the production of GHG. Although, some CO2 is still produced during the manufacture and construction of (RE) devices^15^. ERS create clean and health environment and reduce the carbon emissions^15^.

## Conclusion

Every economic development requires energy as a vital input. Oil, petroleum, coal, and natural gas are the primary non-renewable sources of energy used today, but their availability are declining. In the future, it was determined that RE is going to be a vital field and have been strongly recommended in the field of research and production of its equipment's based on the new trend in the growth of populated areas, taking into account the high ratio of residential activity in energy consumption. The biggest problem with adopting RE is that it is more expensive than fossil fuels. This type of energy produces low net energy ratios, but it is always available. In spite of potential cost reductions from research on this technology, RE costs may not soon be comparable with the cost of fossil fuels. The economic and environmental implications of employing RE sources were the focus of this study. It demonstrates that ERS elements may be used effectively in every city, but it also demonstrates that ERS elements increase the initial cost of the road, and the payback period will take almost 3–4 years. Additionally, ERS elements can generate more energy than the road itself might use. If third-world nations promote the use of ERS elements in their roads, their cities will be able to support their own energy needs and will be able to sell this continuous energy generation for a profit. These underdeveloped nations, which are underdeveloped because of the energy issue, can be developed with the aid of ERS elements. This research is valuable because it provides a brand-new component called ERS elements that can be used anywhere and contributes to addressing major environmental issues like lowering carbon emissions, ozone depletion, and climatic changes.

## Recommendation

The research proposed an integration between streetscape elements with (RE) sources, to result in Enery-RoadScape elements that were proved theoretically to reduce Carbon emissions. However, it is strongly recommended that these elements undergo industrial development processes so that the theory becomes policy.

## Data Availability

The datasets that were collected from the private companies and helped analyse the ERS database are not publicly available due to the request of the companies because it may affect their sales strategy, but they are available from the corresponding author on reasonable request.
